# Interaction between the genomes of *Lactococcus lactis* and phages of the P335 species

**DOI:** 10.3389/fmicb.2013.00257

**Published:** 2013-08-30

**Authors:** William J. Kelly, Eric Altermann, Suzanne C. Lambie, Sinead C. Leahy

**Affiliations:** AgResearch Limited, Grasslands Research CentrePalmerston North, New Zealand

**Keywords:** phage, *Lactococcus lactis* subsp. *cremoris*, dairy, starter culture, genome, integrase, cell wall polysaccharides

## Abstract

Phages of the P335 species infect *Lactococcus lactis* and have been particularly studied because of their association with strains of *L. lactis* subsp. *cremoris* used as dairy starter cultures. Unlike other lactococcal phages, those of the P335 species may have a temperate or lytic lifestyle, and are believed to originate from the starter cultures themselves. We have sequenced the genome of *L. lactis* subsp. *cremoris* KW2 isolated from fermented corn and found that it contains an integrated P335 species prophage. This 41 kb prophage (Φ KW2) has a mosaic structure with functional modules that are highly similar to several other phages of the P335 species associated with dairy starter cultures. Comparison of the genomes of 26 phages of the P335 species, with either a lytic or temperate lifestyle, shows that they can be divided into three groups and that the morphogenesis gene region is the most conserved. Analysis of these phage genomes in conjunction with the genomes of several *L. lactis* strains shows that prophage insertion is site specific and occurs at seven different chromosomal locations. Exactly how induced or lytic phages of the P335 species interact with carbohydrate cell surface receptors in the host cell envelope remains to be determined. Genes for the biosynthesis of a variable cell surface polysaccharide and for lipoteichoic acids (LTAs) are found in *L. lactis* and are the main candidates for phage receptors, as the genes for other cell surface carbohydrates have been lost from dairy starter strains. Overall, phages of the P335 species appear to have had only a minor role in the adaptation of *L. lactis* subsp. *cremoris* strains to the dairy environment, and instead they appear to be an integral part of the *L. lactis* chromosome. There remains a great deal to be discovered about their role, and their contribution to the evolution of the bacterial genome.

## Introduction

The role of *Lactococcus lactis* as the key organism in the initiation of milk fermentation has been known since the work of Joseph Lister in the 1870s when *Bacterium lactis* was the first bacterium to be isolated in pure culture (Santer, 2010). Today this species is the main constituent of cultures used for the manufacture of a vast range of fermented dairy products, including fermented milks, sour cream, soft and hard cheeses, and lactic casein. The taxonomy of *L. lactis* is confused by discrepancies between phenotypic and genotypic descriptions but it is apparent that two subspecies exist that correlate with genotype and are known as *L. lactis* subsp. *cremoris* and *L. lactis* subsp. *lactis* (Kelly et al., 2010). Both subspecies are used as starters by the dairy industry but the strains with the *L. lactis* subsp. *cremoris* genotype cluster closely together (Rademaker et al., 2007), and are particularly favored for use as defined strain starter cultures for Cheddar cheese production.

The use of defined strain starters began in New Zealand in the 1930s using bacteria selected from mixed strain cultures, and studies of bacteriophages that infect dairy cultures began around the same time (Whitehead and Cox, 1936), as fermentations using single strain starters quickly proved to be susceptible to phage attack. Subsequently, phage resistance and the selection and characterization of phage-unrelated strains, became the major focus of dairy starter culture research (Lawrence et al., 1978), and it was concluded that a relatively small number of significantly different *L. lactis* subsp. *cremoris* starter strains exist (Whitehead and Bush, 1957; Lawrence et al., 1978; Kelly et al., 2010). Initial studies investigated whether phages originated from the dairy environment or from the cultures themselves, but eventually the environmental origin came to be regarded as the most significant and a range of measures were developed to control phage attack (Whitehead, 1953). Nevertheless, phage attack, leading to slow or dead vats, remains the leading cause of fermentation problems in the dairy industry. It was also discovered that most starter strains were lysogenized by one or more bacteriophage, and that these temperate phages could be induced, although it was difficult to find strains that they could be propagated on (Huggins and Sandine, 1977; Terzaghi and Sandine, 1981).

Bacteriophages from *L. lactis* were originally divided into 12 species within the *Siphoviridae* and *Podoviridae* based on morphology and DNA homology, and type phages were selected to represent each species (Jarvis et al., 1991). Subsequent studies have refined the number of species to 10 (Deveau et al., 2006), with most of the phage problems encountered in dairy fermentations ascribed to just three species. These are the small isometric-headed 936 and P335 species, and the prolate-headed c2 species. The 936 and c2 species are lytic whereas phages from the P335 species may have a temperate or lytic lifestyle (Braun et al., 1989). Phages of all three species have now had their genomes sequenced and while phages from the 936 and c2 species form distinct but homogeneous groups, the phages from the P335 species are much more heterogeneous and their genomes appear to be mosaic in structure similar to the lambdoid phages. Most studies of phages of the P335 species have focused on their lytic or temperate relationship with *L. lactis* subsp. *cremoris* dairy starter strains. Here we describe a prophage from the genome of a non-dairy *L. lactis* subsp. *cremoris* culture and compare its genome organization with a range of other phages of the P335 species from *L. lactis.*

## Materials and methods

### Genome sequencing

*L. lactis* subsp. *cremoris* KW2 was originally isolated from kaanga wai (fermented corn) and was chosen for sequencing because in studies comparing multiple *L. lactis* cultures (Rademaker et al., 2007) it grouped with the *L. lactis* subsp. *cremoris* dairy starter cultures. Its genome sequence was determined using pyrosequencing of 3 kb mate paired-end sequence libraries on a 454 GS FLX platform with titanium chemistry (Macrogen, South Korea). Pyrosequencing reads were assembled using the Newbler assembler version 2.5.3 (Roche 454 Life Sciences, USA) resulting in 22 contigs across a single scaffold. Gap closure was managed using the Staden package (Staden et al., 2000), and gaps were closed using additional Sanger sequencing by standard PCR-based techniques. Protein-encoding genes were identified by Glimmer (Delcher et al., 1999) and a GAMOLA/ARTEMIS (Rutherford et al., 2000; Altermann and Klaenhammer, 2003) software suite was used to manage genome annotation. Assignment of protein function to ORFs was performed manually using results from BLASTP, and the COG (Clusters of Orthologous Groups, Tatusov et al., 2000), Pfam (Punta et al., 2012), and TIGRFAM (Haft et al., 2013) databases.

### *Lactococcus lactis* genomes

Complete sequences are available for the chromosomes of eight *L. lactis* strains. For *L. lactis* subsp. *cremoris* these are the cheese starter cultures A76 (isolated in France, Bolotin et al., 2012), SK11 (isolated in New Zealand, Makarova et al., 2006) and UC509.9 (isolated in Ireland, Ainsworth et al., 2013), and the plasmid-free reference strain MG1363 (Wegmann et al., 2007). All of these strains originally derive from mixed strain dairy starter cultures. A feature of the three cheese starter cultures is that they harbor several plasmids which enable these strains to metabolize lactose and casein efficiently, and that their chromosomes contain a large number of transposases and pseudogenes. For *L. lactis* subsp. *lactis* there are genomes for the plasmid-free reference strain IL1403 which is of dairy origin (Bolotin et al., 2001), and three non-dairy strains, KF147 (Siezen et al., 2010), CV56 (Gao et al., 2011), and IO-1 (Kato et al., 2012).

### P335 phage genomes

Table 1 lists the phages belonging to the P335 species for which genome information is available. Three genomes are of lytic phages; P335 (Labrie et al., 2008), 4268 (Trotter et al., 2006), and ul36 (Labrie and Moineau, 2002), with partial genome sequence information also available for an additional lytic phage, Φ 31 (Madsen et al., 2001). Six genomes are from temperate phages that have been induced from their host strain and propagated, usually on either a closely related strain (strain H2 for BK5-T) or a prophage-cured derivative (cured derivative of strain R1 for r1t). Publically available temperate phage genomes are: TP901-1 (Brøndsted et al., 2001), Tuc2009 (NCBI Reference Sequence: NC_002703), r1t (van Sinderen et al., 2006), BK5-T (Desiere et al., 2001), Φ LC3 (Blatny et al., 2004), and Φ smq86 (Labrie and Moineau, 2007). All of these phages have been studied because of their ability to lyse dairy starter strains, although most have a relatively narrow host range.

**Table 1:** **Genomes from lytic and induced P335 species phages, and from P335 prophages located in chromosomes of *L. lactis* strains**.

**Phage**	**Status**	**Host^1^**	**Size (kb)**	**Host origin**	**Accession no.^2^**
P335	Lytic	*L. lactis* subsp. *lactis* biovar diacetylactis F7/2	34	Dairy starter	DQ838728
4268	Lytic	*L. lactis* subsp. *lactis* DPC4268	37	Dairy starter	NC_004746
ul36	Lytic	*L. lactis* subsp. *cremoris* SMQ-86	37	Dairy starter	NC_004066
Φ 31 (fragment)	Lytic	*L. lactis* subsp. *lactis* NCK203		Dairy starter	AJ292531
TP901-1	Temperate/Induced	*L. lactis* subsp. *cremoris* 901-1	38	Dairy starter	NC_002747
Tuc2009	Temperate/Induced	*L. lactis* subsp. *cremoris* UC509	38	Dairy starter	NC_002703
r1t	Temperate/Induced	*L. lactis* subsp. *cremoris* R1	33	Dairy starter	NC_004302
BK5-T	Temperate/Induced	*L. lactis* subsp. *cremoris* BK5	40	Dairy starter	NC_002796
Φ LC3	Temperate/Induced	*L. lactis* subsp. *cremoris* IMN-C3	32	Dairy starter	NC_005822
Φ smq86	Temperate/Induced	*L. lactis* subsp. *cremoris* SMQ-86	34	Dairy starter	DQ394810
bIL285	Prophage	*L. lactis* subsp. *lactis* IL1403	36	Dairy starter	AF323668
bIL286	Prophage	*L. lactis* subsp. *lactis* IL1403	42		AF323669
bIL309	Prophage	*L. lactis* subsp. *lactis* IL1403	37		AF323670
SK11-2	Prophage	*L. lactis* subsp. *cremoris* SK11	37	Dairy starter	NC_008527
SK11-3	Prophage	*L. lactis* subsp. *cremoris* SK11	32		
SK11-4	Prophage	*L. lactis* subsp. *cremoris* SK11	40		
t712	Prophage	*L. lactis* subsp. *cremoris* MG1363	42	Dairy starter	NC_009004
MG-3	Prophage	*L. lactis* subsp. *cremoris* MG1363	44		
A76-1	Prophage	*L. lactis* subsp. *cremoris* A76	36	Dairy starter	NC_017492
A76-2	Prophage	*L. lactis* subsp. *cremoris* A76	38		
pp1	Prophage	*L. lactis* subsp. *lactis* KF147	35	Bean sprouts	NC_013656
pp2	Prophage	*L. lactis* subsp. *lactis* KF147	54		
pi1	Prophage	*L. lactis* subsp. *lactis* CV56	35	Human vagina	NC_017486
pi2	Prophage	*L. lactis* subsp. *lactis* CV56	37		
pi3	Prophage	*L. lactis* subsp. *lactis* CV56	34		
ps3	Prophage	*L. lactis* subsp. *lactis* IO-1	36	Kitchen drain	AP012281
Φ KW2	Prophage	*L. lactis* subsp. *cremoris* KW2	41	Fermented corn	CP004884

1 For lytic phages the host is the strain used for phage propagation; for temperate/induced phages the host is the strain the phage was induced from with propagation on a closely related strain or a cured derivative of the host; for prophages the host is the strain containing the integrated phage genome and these have not been propagated.

2 Accession numbers are for the phage genome where that is separately available, or for the bacterial genome containing the integrated prophage.

Seventeen complete prophages were identified in *L. lactis* genomes, and with the exception of UC509.9 (a derivative of UC509 that has been cured of the Tuc2009 prophage, Ainsworth et al., 2013), all of the sequenced *L. lactis* strains have at least one full-length P335 species prophage integrated into their chromosome (Table 1). The prophages from strains IL1403, MG1363, and SK11 have been described in detail previously (Chopin et al., 2001; Ventura et al., 2007). Phage remnants are also found in these chromosomes, but were not included in the analysis as they have been shown to form a separate subgroup (Ventura et al., 2007).

### Functional genome distribution of 26 *Lactococcus lactis* P335 phages

Publicly available phage genomes were downloaded in Genbank format from the NCBI genome database. Similarly, publicly available *Lactococcus lactis* genomes were downloaded in Genbank format, and integrated prophage genomes identified. All phage genomes were then automatically annotated using GAMOLA (Altermann and Klaenhammer, 2003). Predicted ORFeomes of all genomes were subjected to a functional genome distribution analysis (Altermann, 2012) and the resulting distance matrix was imported into MEGA5 (Tamura et al., 2011). The functional distribution was visualized using the UPGMA method (Sneath and Sokal, 1962).

## Results

### *Lactococcus lactis* subsp. *cremoris* KW2 genome sequence

The genome of *L. lactis* subsp. *cremoris* KW2 consists of a 2,427,048 bp circular chromosome, with a G + C content of 36.65%. KW2 has 61 tRNA genes and 2278 coding sequences (CDS) were predicted. The chromosomal gene content of KW2 is highly similar to *L. lactis* subsp. *cremoris* dairy starter strains, but KW2 has no plasmids, and the chromosome has no transposases and few pseudogenes. The genome sequence of *L. lactis* subsp. *cremoris* KW2 has been deposited in the GenBank database under the accession number CP004884.

### Prophage ϕKW2 genome organization

**Figure 1:**
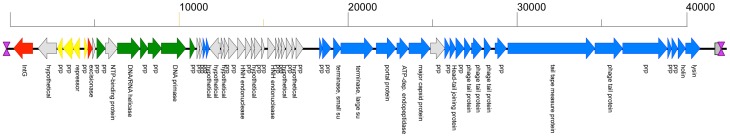
**Genome organization of ϕKW2**. ORFs are drawn to scale and annotations are shown in vertical text. ORFs which show a high level of homology (average of >95% amino acid identity) to ORFs present in other *L. lactis* P335 phage genomes are colored as follows: Red, A76-1; Yellow, bIL312; Green, Φ 31; Blue, MG-3, and other phages belonging to Group 3 from Figure 2. ORFs which match to hypothetical proteins or to other phages are shown in gray. The phage left and right attachment sites are indicated by purple hourglass symbols, respectively. A predicted unspecified tRNA is shown as a gray box. The absolute size of the phage genome is indicated as a horizontal bar above the genome map, and the numbers indicate nucleotide position. prp, phage-related protein; su, sub unit.

The KW2 chromosome contains an integrated 41 kb prophage genome (Φ KW2, kw2_1785 to _1841). The consolidated gene model (Figure 1) shows that Φ KW2 harbors 57 ORFs, for 22 of which a predicted function could be assigned. A further 28 ORFs could be linked to phage-related homologs without a described function. All ORFs are on the same strand except for a single hypothetical protein (kw2_1822) and the ORFs involved in establishment and maintenance of lysogeny. The phage ORFs are flanked by 22-bp sequences indicative of *attL* and *attR* sites. A full list of the predicted proteins that are encoded by the Φ KW2 genome is shown in Table 2.

**Table 2:** **Predicted proteins encoded by the prophage ϕKW2**.

**ORF**	**Annotation**	**Size (aa)**	**Best blast match**	**aa id/sim (%)**
	attR aaaggggcaaaaaaggggcata			
1785	Phage lysin glycoside hydrolase GH25 family	287	MG1363. MG-3 llmg_2082	99/99
1786	Phage holin	150	MG1363. MG-3 llmg_2083	97/98
1787	Phage-related protein	116	MG1363. MG-3 llmg_2084	89/96
1788	Phage-related protein	78	MG1363. MG-3 llmg_2085	92/97
1789	Phage tail fiber protein^1^	881	MG1363. MG-3 llmg_2086	85/92
1790	Phage distal tail protein	548	BK5-Tp16	96/97
1791	Phage tail tape measure protein	1719	MG1363. MG-3 llmg_2089	89/94
1792	Phage-related protein	225	KF147. pp2 pp224	99/99
1793	Phage tail protein	138	MG1363. MG-3 llmg_2090	99/99
1794	Phage tail protein	211	KF147. pp2 pp226	92/94
1795	Phage tail protein	131	KF147. pp2 pp227	98/99
1796	Phage-related protein	168	MG1363. MG-3 llmg_2093	100
1797	Phage head-tail joining protein	116	IL1403. bIL286 Orf47	97/97
1798	Phage-related protein	107	IL1403. bIL286 Orf46	93/97
1799	Phage-related protein	288	IL1403. bIL285 Orf45	92/96
1800	Phage major capsid protein	419	KF147. pp2, pp231	90/95
1801	Phage atp-dependent endopeptidase	234	MG1363. MG-3 llmg_2097	99/99
1802	Phage portal protein	390	MG1363. MG-3 llmg_2098	100
1803	Phage terminase large subunit	636	4268	97/99
1804	Phage terminase small subunit	151	4268	99/99
1805	Phage-related protein	172	BK5-Tp63	99/99
1806	Phage-related protein	58	IL1403. bIL286 Orf38	95/95
1807	Phage-related protein	141	MG1363. t712 llmg_0821	98/100
1808	Hypothetical protein	53	CV56. pi3 CVCAS_2130	92/98
1809	Phage-related protein	115	MG1363. MG-3 llmg_2107	36/63
1810	Hypothetical protein	71	LLCRE1631_01453	93/99
1811	Phage-related protein	60	4268	80/87
1812	Phage-related protein	61	IL1403. bIL309 Orf28	79/87
1813	Phage-related HNH endonuclease family protein	147	*Enterococcus* phage EFRM31	59/69
1814	Phage-related protein	57	*Lactococcus garvieae* DCC43	75/90
1815	Phage-related protein	142	KF147. pp1 pp123	40/57
1816	Hypothetical protein	66	*Lactococcus garvieae* DCC43	90/92
1817	Phage-related protein	117	4268	62/72
1818	Phage-related HNH endonuclease family protein	151	*Enterococcus* phage EFRM31	60/70
1819	Phage-related protein	175	A76. A76-1 llh_3320	50/61
1820	Phage-related protein	93	IL1403. bIL285 Orf26	96/98
1821	Hypothetical protein	60	unknown	
1822	Hypothetical protein	210	*Geobacter metallireducens* GS-15	32/57
1823	Hypothetical protein	63	MG1363. MG-3	95/95
1824	Phage-related protein	65	MG1363. MG-3 llmg_2117	100
1825	Phage-related protein	56	IL1403. bIL285 Orf24	86/89
1826	Phage-related protein	57	IL1403. bIL285 Orf22	84/96
1827	Phage-related protein	103	Φ 31	89/93
1828	Phage-related DNA primase	487	Φ 31	98/99
1829	Phage-related protein	268	Φ 31	98/99
1830	Phage-related protein	150	Φ 31	99/99
1831	Phage DNA/RNA helicase	448	Φ 31	98/99
1832	Phage nucleotide-binding protein	239	*Streptococcus porcinus* str. Jelinkova 176	90/96
1833	Phage-related protein	169	Φ 31	98/99
1834	Phage-related protein	56	IL1403. bIL309 Orf10	95/98
1835	Excisionase	86	A76. A76-1 llh_3245	98/100
1836	Phage-related protein	63	IL1403. bIL312 Orf6	98/100
1837	Phage repressor	122	IL1403. bIL312 Orf5	90/96
1838	Phage-related protein	195	IL1403. bIL312 Orf4	90/95
1839	Phage-related protein	98	IL1403. bIL312 Orf3	99/100
1840	Hypothetical protein	364	*Streptococcus oralis* SK304	59/80
1841	Phage integrase	379	A76. A76-1 llh_3210	99/99
	attL aaaggggcactaaaggggcata			

1 This is much smaller than the corresponding protein predicted from MG-3 (881 vs. 1617 aa).

### Comparison of P335 phage genomes

**Figure 2:**
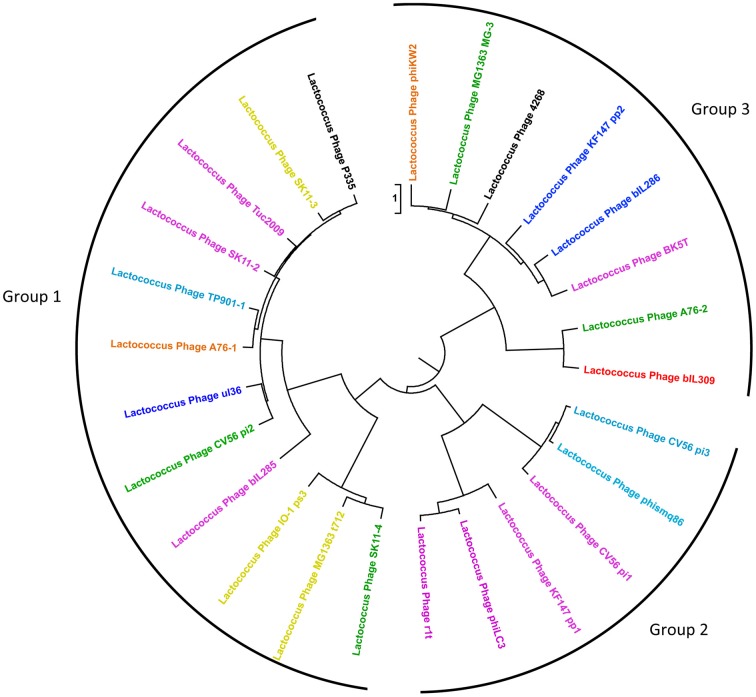
**Functional genome distribution of 26 *Lactococcus lactis* P335 phage genomes**. Comparison of the complete ORFeomes of all P335 species phage and prophage genomes. The tree is drawn to scale, with branch lengths in the same units as those of the functional distances used to infer the distribution tree. Three major clusters were identified and are depicted as Groups 1, 2, and 3. The coloring of individual phage relates to their predicted site for chromosomal integration (see Figure 3) as follows: Red, site 1; Yellow, site 2; Dark blue, site 3; Purple, site 4; Orange, site 5; Green, site 6; Blue, site 7; Black, lytic phage with no integrase gene. Although ul36 is a lytic phage it retains an integrase indicating that it may not have lost the ability to lysogenize. This analysis is based on the position of the prophage in the various *L. lactis* genomes and on the high level of conservation seen between the integrases of the phages that integrate at each specific site.

Functional genome distribution was used to compare the genomes of the phages belonging to the P335 species. The results of this analysis are shown in Figure 2. The 26 phage genomes fall into three broad groups which show a close resemblance to those previously described by others (Trotter et al., 2006; Ventura et al., 2007; Samson and Moineau, 2010). Recently two lytic phages isolated in North America have been sequenced and assigned to a fourth P335 phage group (Mahoney et al., 2013b). The region of the genome devoted to morphogenesis genes is the most conserved (Labrie et al., 2008). From current sequence data the only gene that is conserved among most phages of the P335 species is that for a dUTPase (Labrie and Moineau, 2002). The phage dUTPase is highly conserved at nucleotide and amino acid levels, but differs significantly from the chromosomal *dut* gene. A phage-like dUTPase is found in each prophage from the dairy *L. lactis* subsp. *cremoris* strains but is not part of the Φ KW2 genome, suggesting that its acquisition may be associated with adaptation to the dairy environment. The role of the phage dUTPase is not known, but it is hypothesized that dUTPases are involved in controlling various cellular processes (Penadés et al., 2013).

### Chromosomal integration

**Figure 3:**
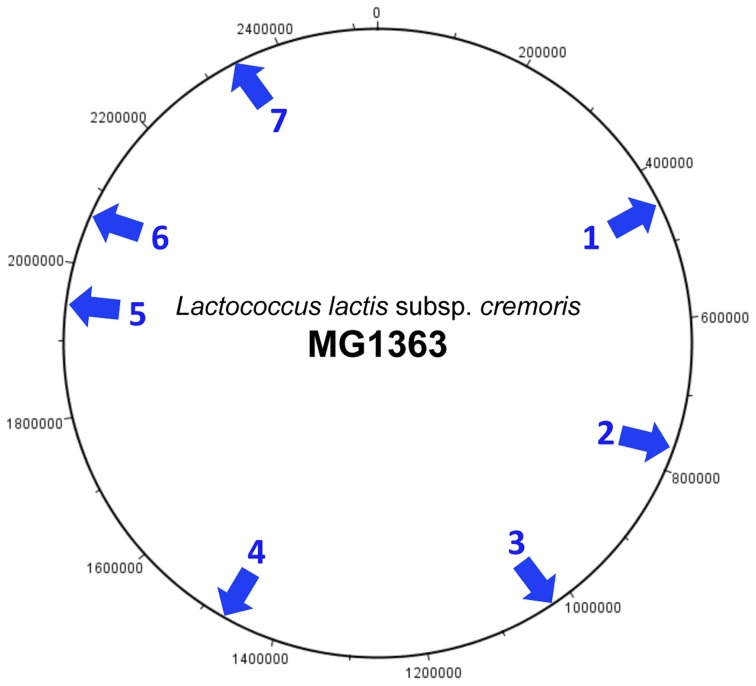
**Integration sites of P335 species prophages on the *L. lactis* subsp. *cremoris* chromosome**. The chromosome of MG1363 is used as a reference. MG1363 is described as a phage- and plasmid-cured derivative of NCDO712, and the genome of the parent strain contains an additional prophage predicted to be integrated at site 7 (Le Bourgeois et al., 2000). The integration sites are located as follows: (1) Adjacent to the tRNA-Arg gene located between llmg_0461 (*lyt*R) and llmg_0462 (*tru*A). (2) Between llmg_0789 (*rbs*B) and llmg_0856. Location of phage t712. (3) Between llmg_1069 and llmg_1079. (4) Between llmg_1514 (*rex*) and llmg_1515 (*rad*C). (5) Between llmg_1968 and llmg_1969 (*suf*B). This site is not present in subsp. *lactis* strains. (6) Between llmg_2081 (*sun*L) and llmg_2143. Location of phage MG-3. (7) Between llmg_2406 (*com*GC) and llmg_2407 (*com*GB).

Analysis of the sequenced *L. lactis* genomes shows that prophage integration is not random but occurs at a limited number of specific sites on the host chromosome. Currently seven different sites have been identified (Figure 3) and each is associated with a highly conserved integrase. The most common location for phage integration is site 4 between the *rex* and *rad*C genes. There is no relationship between the three groups of phages belonging to the P335 species and the integrase that is present (Figure 2).

### Host specificity and phage receptors

Phage infection requires the recognition of receptors on the bacterial cell surface by receptor-binding proteins (RBPs) that are part of the phage tail structure. The RBP from TP901-1 has been characterized and forms part of a complex baseplate structure (Spinelli et al., 2006; Bebeacua et al., 2010; 2013). TP901-1 and the closely related Tuc2009 have similar structural tail proteins, but different host ranges and RBPs (Vegge et al., 2006), and based on their genome sequences other phages of the P335 species are also predicted to have different RBPs. The RBPs are known to bind to carbohydrate cell surface receptors but the exact nature of these remains unknown. However, analysis of the genome of *L. lactis* subsp. *cremoris* KW2 suggests that there are possibly four different cell wall polysaccharides (CWP) synthesized by the cell.

*L. lactis* strains all contain a cluster of >20 genes (kw2_0186-_0208) which together determine the biosynthesis of a CWP that forms a pellicle on the cell surface, and is believed to have a role as a bacteriophage receptor for 936 phages (Mahoney et al., 2013a). Genes encoding the biosynthesis and transfer of rhamnose are located at one end of this gene cluster and appear to be strongly conserved, but the remainder of the genes vary between the different strains. This variability makes this CWP a suitable candidate as a receptor for phages of the P335 species. A second cluster of potential CWP biosynthesis genes is present in KW2 (kw2_2123-_2131), but some of these are pseudogenes in the *L. lactis* subsp. *cremoris* dairy strains, and so this CWP seems an unlikely candidate as a phage receptor as it is probably not produced in all strains.

It is reported that the most likely receptors are believed to be glycerol- or phosphoglycerol-containing wall teichoic acids (WTA) or lipoteichoic acids (LTAs), as their likely variable structures could determine strain specificity (Spinelli et al., 2006). Despite this, little is known about teichoic acid production by *L. lactis*. Structures have been determined for the LTA and extracellular (wall) teichoic acid components of *L. lactis* G121 (Fischer et al., 2011, 2012). This strain was isolated from a cowshed and has been shown to have allergy-protective properties, but the teichoic acids are likely to be different to those from the *L. lactis* subsp. *cremoris* dairy starter strains used in most phage studies.

Analysis of *L. lactis* genomes shows that genes for the biosynthesis of WTA occur as a large cluster in the plant *L. lactis* strain KF147 (Siezen et al., 2011) and also in KW2 (kw2_0901 to _0922). The genes at either end of the cluster which encode the assembly and export functions are conserved but the remaining genes, presumed to determine the sugar composition, differ. The situation is drastically different with the *L. lactis* subsp. *cremoris* dairy starter strains. In MG1363 these central genes have been replaced by transposases while in SK11, A76, and UC509.9 only the genes at the ends of the cluster remain. It may be concluded that these dairy strains no longer produce WTA, and it is unlikely to be the phage receptor. How this loss of WTA contributes to the phage resistance phenotype of dairy strains remains to be determined.

Genes encoding the biosynthesis of LTA have been determined for some Gram positive bacteria (Reichmann and Gründling, 2011) but have not been characterized in *L. lactis*. However, a lipoteichoic acid synthase (LtaS), and two glycolipid biosynthesis glycosyltransferases (LafA and LafB) have been identified in the A76 genome and these are conserved in the other *L. lactis* subsp. *cremoris* strains including KW2 (kw2_0755, and kw2_2197-_2198). Other genes involved in biosynthesis of the carbohydrate moieties of the LTA remain to be identified. These strains also have the *dlt*A-D operon which mediates the D-alanylation of LTA and contributes to the cell surface properties (Giaouris et al., 2008). Consequently LTA is a candidate for a cell surface receptor likely to be recognized by phages of the P335 species, although it appears to lack the necessary strain variability. Consequently accurate identification of the host receptor awaits further study.

### Phages of the P335 species as agents of gene transfer and chromosomal rearrangement

Transduction of genetic information by temperate phages has been described for lactococci and used to study plasmid-associated lactose and proteinase phenotypes in NCDO712 and related strains (Gasson, 1990). Recently, Wegmann et al. (2012) used plasmid transduction by the Φ TP712 (t712) phage in their analysis of the pLP712 plasmid from NCDO712, but otherwise transduction has been seldom used with *L. lactis*. There is no information available on transduction by temperate phages from other lactococcal strains, and consequently their role in horizontal gene transfer is not known. Lysogenic conversion can be viewed as a type of specialized transduction, and in the Gram positive pathogens *Staphylococcus aureus* and *Streptococcus pyogenes*, lysogenic conversion genes involved in virulence are found located distal to the phage lysin. Examination of the prophages from the dairy *L. lactis* subsp. *cremoris* strains suggests that lysogenic conversion is not common. However, the t712 prophage found in MG1363 does have an abortive infection gene located between the lysin and *attR* site (Ventura et al., 2007).

Analysis of the *L. lactis* subsp. *cremoris* genomes shows that P335 species prophages can be involved in mediating significant changes to chromosomal architecture. In *L. lactis* subsp. *cremoris* A76 the prophage integration sites 1 and 6 are found at the ends of a chromosomal rearrangement. The chromosome of strain A76 has truncated integrase, repressor and phage lysin genes adjacent to the *lyt*R gene (llh_2555, site 1). This region also contains several transposases and then joins to the ribosomal RNA small subunit methyltransferase B gene (*sun*L, llh_2615, site 6). Phage A76-2 is located at the other end of the chromosomal rearrangement. The phage integrase (llh_10885) is adjacent to the A76 homolog of llmg_2143 (llh_10890, site 6), while the lysin gene (llh_10615) is separated from *tru*A (llh_10580, site 1) by a transposase and other hypothetical proteins. A similar situation possibly applies with other *L. lactis* subsp. *cremoris* strains HP and FG2 (Kelly et al., 2010) that show similar chromosomal rearrangements to strain A76.

## Discussion

Phages that attack *Lactococcus lactis* dairy starter cultures remain the largest single cause of industrial milk fermentation problems and result in significant economic losses. While most problems result from infection by virulent 936 and c2 species phages, (Madera et al., 2004), phages of the P335 species are also of interest as they likely originate from the starter cultures themselves. However, it is not clear what proportion of significant problems result from the phages of the P335 species. While some P335 phages have adopted a lytic lifestyle, it is possible that this is a relatively rare event, as the lytic P335 phages have a narrow host range (Moineau et al., 1996), and it has proved difficult to find propagation hosts for temperate phages that have been induced from *L. lactis* dairy strains. Nevertheless, Labrie and Moineau (2007) have clearly shown how a lytic P335 phage can exchange functional modules with an integrated prophage to rapidly generate novel combinations with altered properties.

Most studies of phages belonging to the P335 species have been in the context of their association with dairy strains of *L. lactis* subsp. *cremoris*, and here we report the genome sequence of *L. lactis* subsp. *cremoris* KW2 that was isolated from fermented corn. The most notable feature of the KW2 chromosome is that it does not show the high numbers of transposases and pseudogenes characteristic of *L. lactis* subsp. *cremoris* dairy starters, but it does contain a single integrated prophage (Φ KW2). Phage evolution occurs by the rearrangement of functional modules, and it seems clear that phages are able to mix-and-match these modules to produce a variety of different combinations. Φ KW2 serves as an example of this as its mosaic structure appears little different from the prophages found in dairy starters, and its various functional modules show very high homology to known dairy phages. The integrase and excisionase (which both match to the A76-1 prophage), the early gene control module including the phage repressors (matches to the P335 phage remnant bIL312 found in the IL1403 genome), and the DNA replication module (matches to the replication genes from the lytic phage Φ 31) are located at one end of the genome. The proteins predicted to be encoded by the central region of the genome show only weak homology mainly to proteins from various phage genomes, and this is typical of other phages from the P335 species. The remaining three modules for DNA packaging, phage structural components (including head and tail morphogenesis and host specificity), and cell lysis are found at the other end of the genome and almost all of the genes in this region show high homology to phage MG-3 from the MG1363 genome and to other phages that make up Group 3 in Figure 2.

The ability of phages of the P335 species to shuffle their various modules means that no *L. lactis* subsp. *cremoris* strains can be said to be truely phage-unrelated. The integrases are a good example as they enable phages to insert at a limited number of specific sites and yet can be partnered with a diversity of different modules as shown in Figure 2. Using conserved primer sequences from four P335 phages that insert at integration site 4 (Tuc2009, r1t, BK5-T, and Φ LC3), O'Sullivan et al. (2000) showed that 19 of 31 cheese-making lactococcal strains gave a product of the expected size indicating the presence of a specific integrase gene, and by implication a prophage inserted at this location. The lytic behavior of these strains could be correlated with the presence of this prophage sequence.

It can be concluded from this that P335 species prophages are an integral part of the *Lactococcus lactis* genome, and their interaction can take several different forms. The prophage may be induced from the genome and in some cases become a lytic phage that is unable to relysogenize. Prophages may also mediate chromosomal rearrangement as appears to be the case for *L. lactis* subsp. *cremoris* A76, or acquire lysogenic conversion genes that modify bacterial properties. In the case of dairy starter cultures prophages may also influence important industrial properties of the strains such as phage resistance and autolysis. Overall it is difficult to quantify the effect of prophage genomes, and it appears most likely that they have co-evolved together with the bacterial genome. The observation that many of the prophages in dairy strains of *L. lactis* subsp. *cremoris* contain transposases suggests that prophage genomes are exposed to the same selection as the host genome, and that prophages may have had only a minor role in the adaptation of *Lactococcus lactis* strains to the dairy environment.

### Conflict of interest statement

The authors declare that the research was conducted in the absence of any commercial or financial relationships that could be construed as a potential conflict of interest.
